# RETRACTION: Zebularine Enhances Apoptosis of Human Osteosarcoma Cells by Suppressing Methylation of ARHI

**DOI:** 10.1111/cas.16346

**Published:** 2024-09-16

## Abstract

**RETRACTION**: K. Ye, S. Wang, J. Wang, H. Han, B. Ma and Y. Yang, “Zebularine Enhances Apoptosis of Human Osteosarcoma Cells by Suppressing Methylation of ARHI,” *Cancer Science* 107, no. 12 (2016): 1851–1857, https://doi.org/10.1111/cas.13088.

The above article, published online on 29 September 2016 in Wiley Online Library (wileyonlinelibrary.com), has been retracted by agreement between the journal Editor‐in‐Chief, Masanori Hatakeyama; the Japanese Cancer Association; and John Wiley & Sons Australia, Ltd. The retraction has been agreed following an investigation into concerns raised by a third party, which revealed that the western blots presented in figure 4a and 4d contain duplicated bands. Furthermore, duplications were found between bands within figure 4d itself, some of which appear to have been horizontally flipped. The authors did not respond to the concerns raised and did not provide their original data. The editors consider the results and conclusion reported in this article unreliable.

**RETRACTION**: K. Ye, S. Wang, J. Wang, H. Han, B. Ma and Y. Yang, “Zebularine Enhances Apoptosis of Human Osteosarcoma Cells by Suppressing Methylation of ARHI,” *Cancer Science* 107, no. 12 (2016): 1851–1857, https://doi.org/10.1111/cas.13088.

The above article, published online on 29 September 2016 in Wiley Online Library (wileyonlinelibrary.com), has been retracted by agreement between the journal Editor‐in‐Chief, Masanori Hatakeyama; the Japanese Cancer Association; and John Wiley & Sons Australia, Ltd. The retraction has been agreed following an investigation into concerns raised by a third party, which revealed that the western blots presented in figure 4a and 4d contain duplicated bands. Furthermore, duplications were found between bands within figure 4d itself, some of which appear to have been horizontally flipped. The authors did not respond to the concerns raised and did not provide their original data. The editors consider the results and conclusion reported in this article unreliable.

